# A Novel Algicide: Evidence of the Effect of a Fatty Acid Compound from the Marine Bacterium, *Vibrio* sp. BS02 on the Harmful Dinoflagellate, *Alexandrium tamarense*

**DOI:** 10.1371/journal.pone.0091201

**Published:** 2014-03-13

**Authors:** Dong Li, Huajun Zhang, Lijun Fu, Xinli An, Bangzhou Zhang, Yi Li, Zhangran Chen, Wei Zheng, Lin Yi, Tianling Zheng

**Affiliations:** 1 State Key Laboratory of Marine Environmental Science and Key Laboratory of MOE for Coast and Wetland Ecosystems, School of Life Sciences, Xiamen University, Xiamen, China; 2 College of Chemical Engineering, Huaqiao University, Xiamen, China; University Paris South, France

## Abstract

*Alexandrium tamarense* is a notorious bloom-forming dinoflagellate, which adversely impacts water quality and human health. In this study we present a new algicide against *A. tamarense*, which was isolated from the marine bacterium *Vibrio* sp. BS02. MALDI-TOF-MS, NMR and algicidal activity analysis reveal that this compound corresponds to palmitoleic acid, which shows algicidal activity against *A. tamarense* with an EC50 of 40 μg/mL. The effects of palmitoleic acid on the growth of other algal species were also studied. The results indicate that palmitoleic acid has potential for selective control of the Harmful algal blooms (HABs). Over extended periods of contact, transmission electron microscopy shows severe ultrastructural damage to the algae at 40 μg/mL concentrations of palmitoleic acid. All of these results indicate potential for controlling HABs by using the special algicidal bacterium and its active agent.

## Introduction

HABs caused by blooms of toxic microalgae, can result in a significantly negative impact on public health and natural resources [1]. As a result of global climate change and the increased pollution of water bodies, HABs have received increasing attention in recent years [2]; in particular, blooms of the toxic species of *Alexandrium* [3]. *Alexandrium tamarense* is a notorious toxic species of *Alexandrium*, which can lead to serious economic losses, human illnesses and even death as a result of the production of paralytic shellfish poison [4], [5]. Several studies show that the blooms can be terminated by rapid cell lysis, which causes a decline in the population until the next bloom [6]–[9]. In an effort to develop short-term solutions for controlling HABs, several approaches are being explored, including chemical methods [10]–[12], physical manipulation (clays, flocculants et al.) [13], [14] and currently, biological agents [15], [16]. Although effective in controlling blooms, chemical approaches are considered to be potentially dangerous since chemical agents can cause serious secondary pollution [17], and can indiscriminately kill other organisms in the aquatic ecosystem, altering marine food webs and eventually impact natural fish communities [18]. While the high cost of physical manipulation may be impractical [19]. Therefore, biological agents, including bacteria [20], protozoa [18], viruses [21] and macrophytes [22] are now being considered as potential suppressors in controlling the outbreak of algal blooms. This has become a research hotspot in recent years based on the advantages of its efficiency, species-specificity and environment-friendliness [23], [24].

Bacteria play an important role in nutrient regeneration and energy transformation in aquatic ecosystems [17]. However, in the past decade or so, studies have revealed the existence of bacteria capable of inhibiting or degrading algal blooms in marine and freshwater environments [8], [25], [26]. Most algicidal bacteria isolated from natural environments to date have been assigned to the Cytophaga, Flavobacterium and Bacteroidetes group (which includes *Cytophaga*, *Saprospira*, *Flavobacterium* and *Zobellia*) or to the γ-proteobacteria group (which includes *Alteromonas*, *Pseudomonas*, *Pseudalteromonas* and *Vibrio*) [25], [27]. These algicidal bacteria appear to be effective through direct or indirect attack on the target algal species, the former requires cell-to-cell contact, while the latter involves the secretion algicidal substances. So far, studies of the relationships between algae and bacteria have focused on mainly the isolation, identification or characterization of the algicidal bacteria [28]–[30]. However, a few studies have focused on the isolation of algicidal compounds from the algicidal bacteria and, although some algicidal substances are reported to be secreted by marine microorganisms, not many such algicidal substances have yet been identified [20], [30]–[32].

We previously reported an algicidal bacterium *Vibrio* sp. BS02, exhibits strong activity against the toxic dinoflagellate *A. tamatense*, which was isolated from a mangrove area in Zhangjiangkou, Fujian Province, China. Alga-lysing characterization of this bacterium suggested that the algicidal activity was due to an extracellular bioactive compound, and the dialysis analysis of the bacterial culture showed that the molecular weight of the algicidal substance is less than 0.5 kD [33]. In our present study we conducted a detailed investigation of t *Vibrio* sp. BS02, and our results demonstrated that the algicidal substance secreted by the BS02 was a fatty acid (the “bioactive compound”), the activity of which is c species-specific. In addition, we studied the ultrastructural changes of the algae caused by the fatty acid and discuss the mechanism of algal cell lysis.

## Materials and Methods

### Bacterial Cultures

BS02 was cultured in marine agar 2216E (pH 7.4∼7.8) at 25°C for 24∼48 h, and was preserved at −80°C in marine broth with 20% (v/v) glycerol. Subculturing was performed in improved actinomyces medium AC1 (20 g soluble starch, 1 g NaNO_3_, 0.5 g K_2_HPO_4_, 0.5 g MgSO_4_ ⋅7H_2_O, 0.01 g FeSO_4_⋅7H_2_O, 75 μg K_2_Cr_2_O_7_ in 1L of 0.45 μm Millipore-filtered seawater) at 25°C and 150 rpm. After 24 h, the bacterial culture was centrifuged at 10,000×g for 10 min to remove the cell debris, and the supernatant was filtered through 0.22 μm polycarbonate filters to obtain a cell-free filtrate, and then stored at 4°C for the experiments.

### Algal Cultures

The *A. tamarense* (ATGD98-006) algal was provided by the Institute of Hydrobiology, Jinan University, Guangzhou, China, in addition, *A. tamarense* DH01(AT), *Alexandrium minutum* (AMTW), *Cyanobacteria* (BA), *Dunallella salina* (DS), *Chlorella autotrophica* (CA), *Heterosigma akashiwo* (HA), *Chattonella marina* (CM), *Phaeodactylum tricornutum* (PT), *Asterionella japonica* (AJ), *Nannochloropsis* sp.(NC) and *Phaeocystis globosa* (PG) were obtained from the State Key Laboratory of Marine Environmental Science in Xiamen University of China. All Cultures were maintained in f/2 medium (without silicate) prepared with natural seawater [34] at 20±1°C under a 12∶12 h light–dark cycle with a light intensity of 50 mmol photons m^−2^s^−1^. Exponential phase axenic cultures were aliquoted for further experiments.

### Assays for Algicidal Activity

The algicidal activity was carried out in 24-well plates (2 mL of *A. tamarense* cultures in the exponential growth phase were assigned to each well). The extracted fractions or purified components dissolved in Dimethyl sulfoxide (DMSO) were added into *A. tamarense* cultures at different final concentrations in triplicate. AC1 broth or DMSO was added to the wells as a control with the same final concentration. Algal growth was monitored every day and the cells were counted using microscopy with a hemocytometer. The percentage growth inhibition was calculated using the following equation [30]:
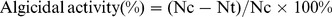



Nc represents the number of algal cells in the control group; and Nt represents the number of algal cells in the treatment group.

### Extraction of Algicidal Compounds

The previous report suggests that algicidal compounds of BS02 strain was extracellularly produced, less than 0.5 kD in molecular weight, as well as non proteinaceous. In order to extract the algicidal compounds, BS02 was prepared in distilled water from cultures grown on AC1 solid medium, was used to inoculate 1000 mL flasks, containing 500 mL AC1 liquid medium. The pre-culture (incubated at 28°C for 1 day in an orbital incubator set to 150 rpm) was used to inoculate (5% v/v) a total volume of 25 L culture medium having the same composition as the pre-culture. The culture broth was centrifuged at 10000×g for 20 min after 3 days’ incubation at 28°C and 150 rpm. The thallus material was collected and extracted three times with ethyl acetate (EA) at room temperature. The supernatant was collected by vacuum concentration, then extracted with an equal volume of EA three times at room temperature. The above EA soluble fractions were collected by evaporating to dryness in vacuo at 35°C. Salt and protein in the crude extract were removed using ethanol precipitation three times. Finally, the residues (saved at 4°C) were dried and weighed, and then dissolved in EA using as small a volume as possible, and subjected to silica gel column chromatography. The silica gel (granularity: 200–300 mesh, pH: 6–7, Shanghai, China), weighing 70 times as much as the dried mixture, was used to fill the glass columns (15×180 mm, 20×200 mm, BOMEX, Beijing, China), and was then eluted with 100 mL dichloromethane followed by 100 mL volumes of a 10% step increase in the amount of EA in the dichloromethane – EA mix at a flow rate of 1 mL/min until pure EA was reached. Fractions of 2 mL per collection tube were combined using thin layer chromatography (TLC, silica gel G, obtained from Qingdao Marine Chemical Factory, Qingdao, P. R. China.). The dried residues were each resuspended in 2 mL of appropriate solvent and 30 μL volumes used to test for algicidal activity as described above. The active component was collected, dried and weighed, subjected to silica gel column chromatography again, and then eluted using a series of volumes of petroleum hexane and EA (4: 0.5; 4: 1.5; 4: 2.5; 4: 3.5, v/v). Fractions of 2 mL per collection tube were collected and the algicidal activity tested on each. 30 g of gel (Sephadex™ LH-20, 30×500 mm, Healthcare Bio-Science AB, Sweden) was used to further purify the active fraction with methanol as the solvent. All of the fractions were collected separately based on the analysis of TLC (GF254, pH: 6.2–6.8, Qingdao, China.) and then dried and weighed.

Algicidal bioassays of each fraction were determined in 24 well plates described above. The remainder of each fraction was stored at −20°C for further analysis.

### Identification of Compounds in the Active Fractions

**Figure 1:**
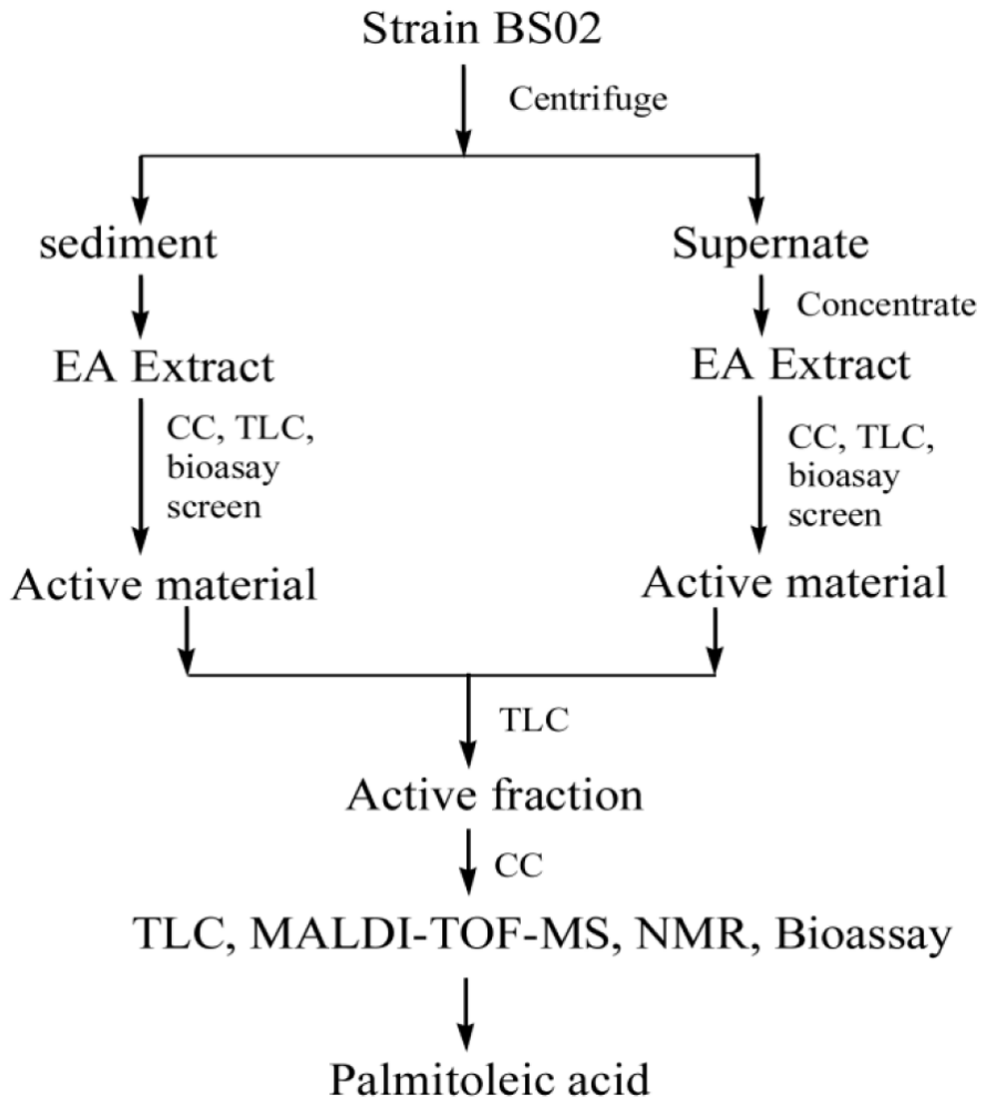
Flow chart for the isolation and identification of the anti-algal compound from Strain BS02. (EA, ethyl acetate; CC, column chromatography; TLC, the thin layer chromatography).

Each algicidal fraction was analyzed using matrix-assisted laser desorption/ionization time of flight mass spectrometry (MALDI-TOF-MS) in order to determine the molecular weight of the algicidal substance. In brief, 0.8 μL of the purified active fraction was placed on the ground board and carbon nanotubes were used as the assistant matrix. The mass spectra were then achieved using a Bruker REFLEX III mass spectrometer (Bruker, Karlsruhe, Germany). Nuclear magnetic resonance (NMR) spectra of the purified algicidal compound were recorded in CDCl_3_ using a DRX500 instrument (Bruker Biospin, Co., Karlsruhe, Germany) at 25°C, and trimethylsilyl (TMS) as the internal standard. A flow diagram showing the full procedure is in Fig. 1.

### The Activity of the Algicidal Compound

Commercially produced algicidal compound was purchased from J&K Scientific Ltd. (Beijing, China) and 0.1 g of the standard compound was dissolved in 1 mL of DMSO to obtain an initial concentration of 100 μg/μL. The algicidal effect of the compound against *A. tamarense* was investigated using a series of concentrations (10, 20, 40, 60 and 80 μg/mL), which were inoculated into 100 mL flasks containing 20 mL of medium with 10^5^ algal cells/mL as the initial algal density. The same volume of the DMSO and AC1 medium (at a concentration of 80 μg/mL) were added to the algal culture as to the negative control. There were three replicates for each concentration and control group. All flasks were cultivated at 20±1°C under a 12∶12 h light-dark cycle with an illumination of about 50 μE⋅m^−2^⋅s^−1^. The viability of the algal cells was monitored under a light microscope (10×40) each day until the fifth day. In addition, the bacterial supernatant (cultivated for 24 h) was added to the *A. tamarense* cultures to a final concentration of 1.0% (v/v) and used as the positive control.

### Assay of the Effect of Palmitoleic Acid on Other Algal Species

The tests of the algicidal effect of palmitoleic acid on the other algal species were conducted for comparison with the results in the literature [22]. First, the algae were cultivated in 24 well plates with 2 mL of algal culture in each well, under the conditions described above. When the cultures reached the logarithmic phase, with a cell density more than approximately 1.0×10^4^ cell/mL, the palmitoleic acid solution (100 μg/μL) was added to the cultures to give a palmitoleic acid final concentration of 40 μg/mL, and the same volume of DMSO was added to the control. Tests were performed in triplicate, and the test plates were cultivated under the conditions described above. After 24 h, the cell densities of the algal cultures were monitored and compared to the control cultures of each species. Non-lysed cultures were considered to be resistant to palmitoleic acid, otherwise they were considered to be weakly or strongly susceptible.

### Submicroscopic Structural Changes in the Algal Cells

*A. tamarense* was incubated for five days in the presence of palmitoleic acid at a final concentration of 40 μg/mL and 1.0% (v/v) f/2 medium. Ten mL aliquots of the culture medium were withdrawn according to the vitality of the algal cells during the first day and then once per day until the fifth day. 0.5 mL portion of the treated cells were fixed with 2.0% glutaraldehyde at 4°C, buffered at pH7.4 in 0.1 M sodium phosphate, then post-fixed with 2.0% O_S_O_4_ in the same phosphate buffer. They were then dehydrated with stepwise increments of ethanol concentrations from 10.0% to 100.0%, and embedded in araldite resin. Ultrathin sections were prepared (*t* = 80–90 nm) using an ultramicrotome, stained with uranyl acetate and lead citrate, each for 15–30 min, then examined using a JEM2100HC electron microscope (JEOL Ltd., Tokyo, Japan) operated at 100 kv.

## Results

### Extraction of Algicidal Compounds

Algicidal activity against *A. tamarense* was found mainly in the EA fraction of the initial *Vibrio* sp. BS02 cell extracts (data not shown). Seven main fractions (A_1_∼A_7_) were collected from the first silica gel chromatography for TLC and bioassay screening. The growth inhibitory effects of these fractions on *A. tamarense* are shown in Table 1. The results suggest that fractions A_2_ and A_3_ exhibit the strongest growth inhibition. The A_2_ and A_3_ fractions were mixed because the TLC analysis suggested the two fractions had similar composition (data not shown). Four further fractions (B_1_∼B_4_) were obtained from the second silica gel chromatography. From Table 1, we know that fraction B_2_ showed the greatest algicidal effect on *A. tamarense*. After the gel chromatography analysis, an oily white fraction, C_1_, was obtained from fraction B_2_, which was considered to be the primary anti-algal substance. This C_1_ fraction was identified using MALDI-TOF-MS and NMR.

**Table 1:** Algicidal activity of the isolated fractions (40 μg/ml) from *Vibrio* sp. BS02 on *A. tamarense* after 3 days incubation.

Fraction	Growth inhibition (%)	Fraction	Growth inhibition (%)
A_1_	12.31±4	B_1_	0±5
A_2_	84.36±3	B_2_	76.98±3
A_3_	78.98±1	B_3_	41.93±12
A_4_	1.37±5	B_4_	27.65±8
A_5_	11.29±6	C_1_	70.21±5
A_6_	25.20±2		
A_7_	42.53±12		

### Identifications of Compound in the Algicidal Fractions

**Figure 2:**
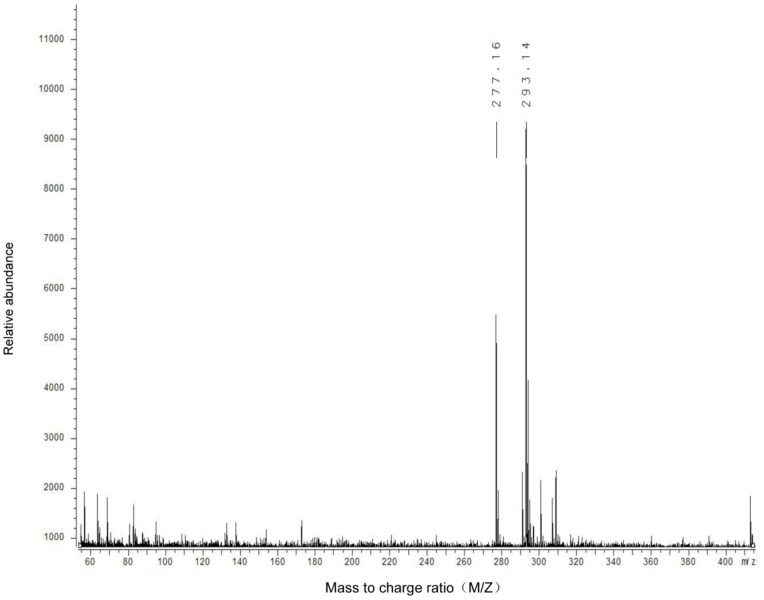
Mass spectrum of the C_1_ fraction obtained from MALDI-TOF MS.

**Figure 3:**
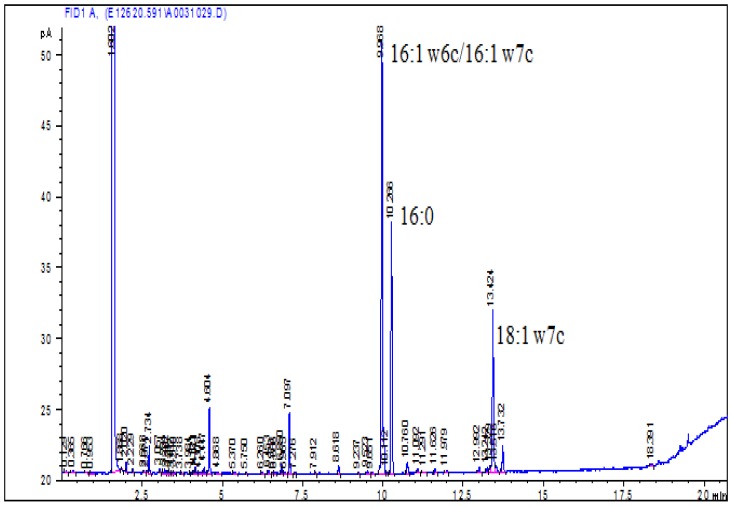
Gas chromatography of the C_1_ fraction.

The MALDI-TOF MS spectrum of the C_1_ revealed two pseudomolecular ions at 277.16 m/z of [M+Na]^+^ and at 293.14 m/z of [M+K]^+^ (Fig. 2). This showed that the molecular weight of the C_1_ fraction was approximately 254. The C_1_ fraction (dried mass  = 9.4 mg) was deemed sufficiently pure using ^1^H-NMR for structural characterization, and the NMR data are shown in Table 2. The ^1^H-^13^C HMBC spectrum enable a full correlation of the triplet in ^1^H-NMR (δ  = 2.35 ppm) with the ^13^C resonance at 179.81 ppm, which clearly confirmed that the compound was a fatty acid. Furthermore, the relative integral intensities of ^1^H resonances indicated a monounsaturated compound with 16-carbon chain. The ^13^C-NMR and DEPT (600 MHz, *δ* ppm from TMS in CDCl_3_) spectrum of C_1_ showed 16 signals: 1 CH_3_ (at *δ* (C) = 14.10 ppm), 12 CH_2_ (at *δ* (C) = 34.00, 31.79, 29.75, 29.69, 29.15, 29.07, 29.04, 29.00, 27.26, 27.17, 24.68 and 22.67 ppm), 2 CH (at *δ* (C) = 129.74, 130.03 ppm), and one carboxyl group at *δ*(C) = 179.81 ppm. The ^1^H-NMR spectrum showed one methyl group signal at δ 0.88 ppm (t, *J* = 6.80 HZ, 3H), and several methylene signals (Table 2). The coupling relationship of ^13^C and ^1^H atoms were further corroborated using ^1^H,^1^H-COSY and HMBC analysis. Comprehensive consideration of the related data of the ^1^H and ^13^C-NMR spectra, DEPT, 2D COSY, HMQC, HMBC and NOESY, compared with the other data evaluated, revealed that the C_1_ fraction was palmitoleic acid (cis-C16∶1 n-7) or palmitelaidic acid (trans-C16∶1 n-7), and the molecular formula was C_16_H_30_O_2_. Another approach to identify whether the compounds was palmitoleic acid or palmitelaidic acid, involved the use of a gas chromatograph (6850; Agilent), and peaks were identified with MIDI software (version 6.0). The result, which is more certain, revealed a monounsaturated 16-carbon chain. (Fig. 3).

**Table 2:** 1H and ^13^C-NMR data °btained in CDCl_3_ at 600 MHz for the algicidal fractions.

δ (ppm)	Integral intensity	Multiplicity	*J* (HZ)	Assignment
^1^H-NMR data				
0.88	3H	t	6.80	H16
1.27–1.34	16H	m	–	H4,H5,H6,H7,H12,H13,H14,H15
1.63	2H	a.q	7.10	H3
2.01–2.03	4H	–	–	H8,H11
2.35	2H	t	7.50	H2
5.33–5.36	2H	m	–	H9,H10
^13^C-NMR data				
14.10	–	s	–	C16
22.67–31.78	–	–	–	C4,C5,C6,C7,C12,C13,C14,C15
24.67	–	s	–	C3
27.25	–	s	–	C8,C11
34.00	–	–	–	C2
129.74	–	d	–	C9,C10
179.81	–	s	–	C1

δ chemical shift, *J* coupling constant, t triplet, a.q apparent quintet, m multiple.

### Biological Activity of the Algicidal Compounds

**Figure 4:**
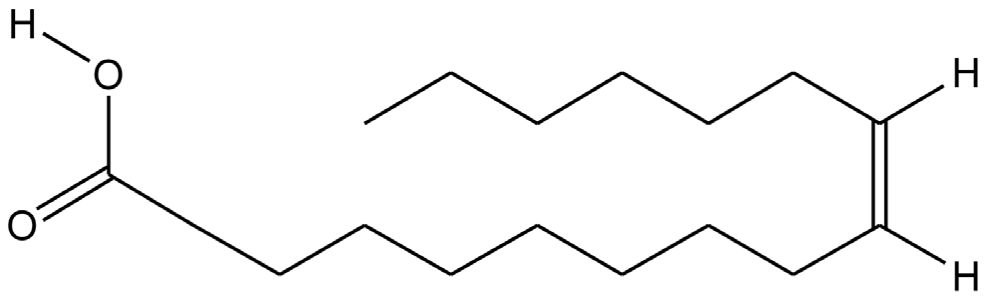
Structure of palmitoleic acid.

**Figure 5:**
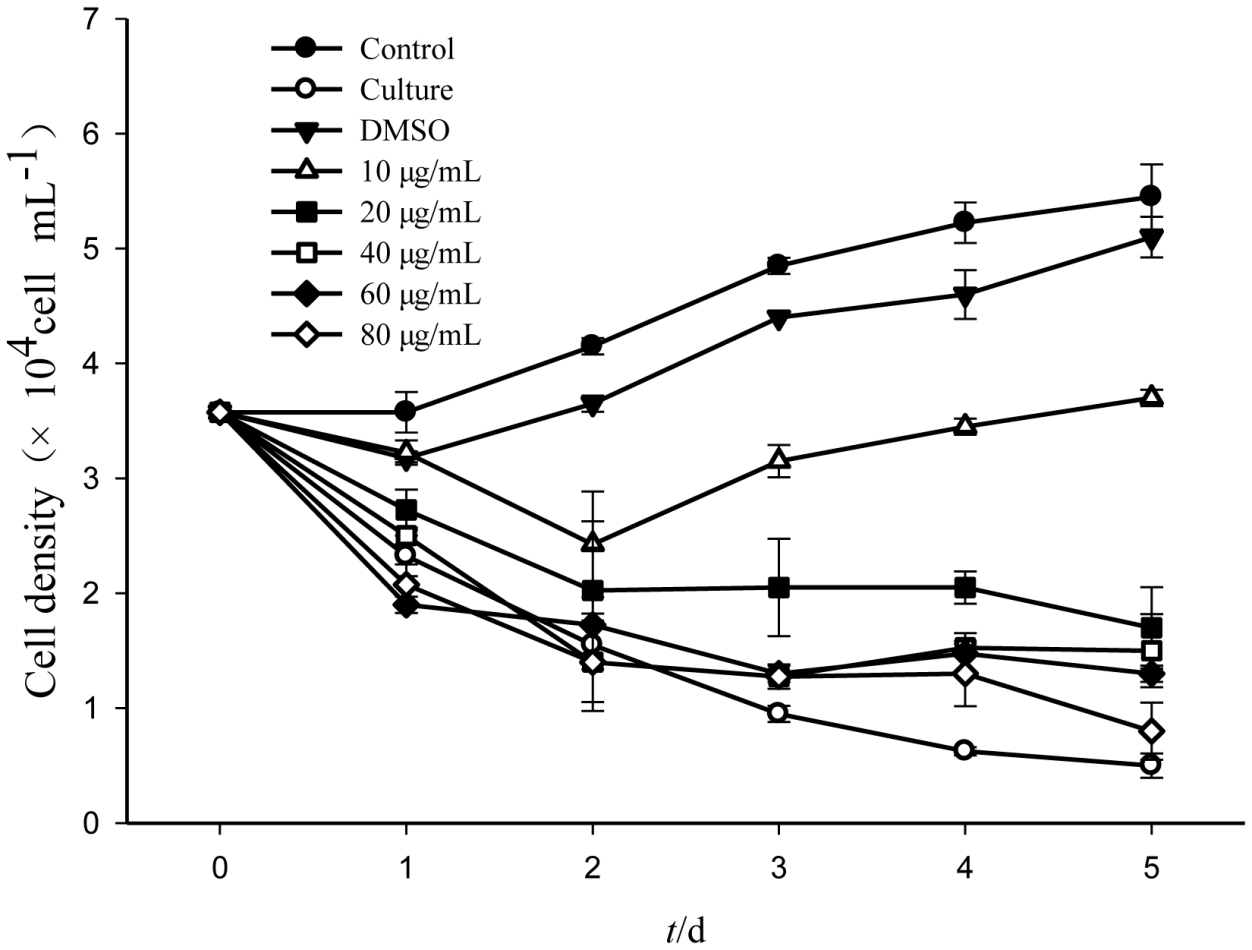
Algicidal effects of the different concentrations of palmitoleic acid on *A. tamarense*. (Control, the Ac1 medium was added to the algal cultures; Culture, the bacterial supernatant of the BS02 strain was added to the algal cultures; DMSO, the dimethyl sulfoxide was added to the algal cultures).

**Figure 6:**
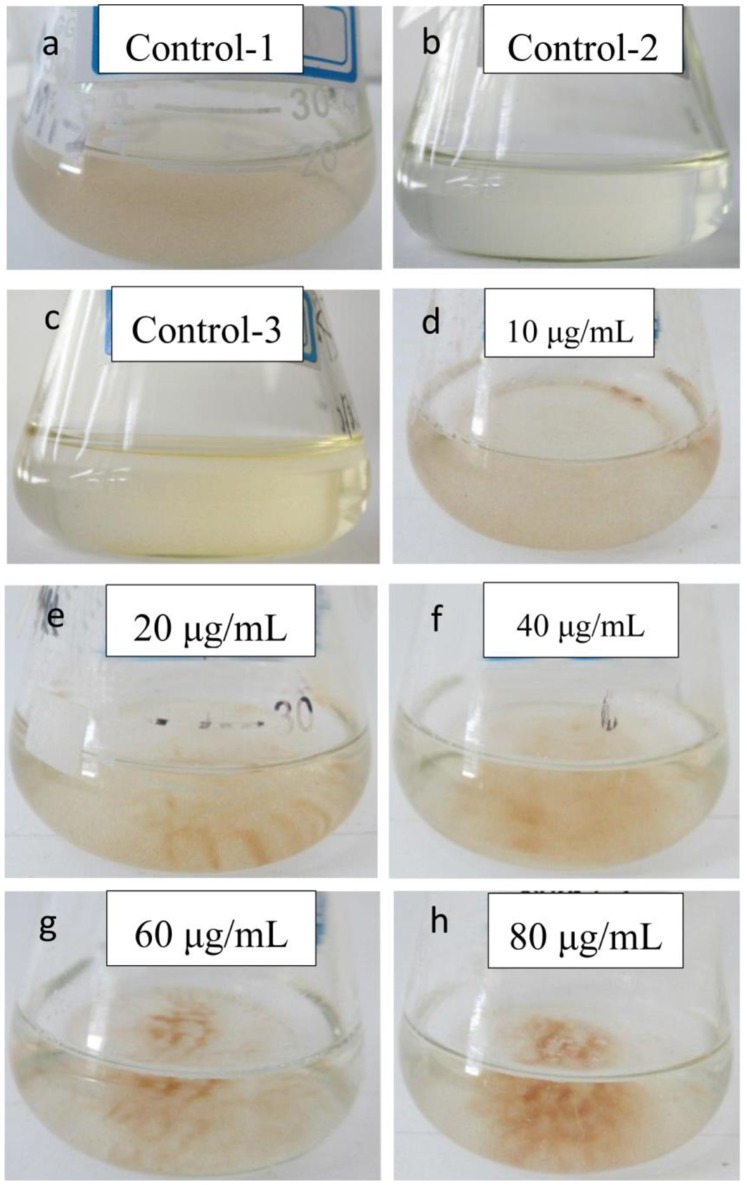
Algicidal effects of the different concentrations of palmitoleic acid on *A. tamarense* after one day. (Control-1, the dimethyl sulfoxide was added to the algal cultures; Control-2, the bacterial supernatant of the BS02 strain was added to the algal cultures; Control-3, the AC1 medium was added to the algal cultures.).

In order to determine whether the effective compound was palmitoleic acid (cis-C16∶1 n-7), palmitelaidic acid (trans-C16∶1 n-7), commercially produced palmitoleic acid, palmitelaidic acid and palmitic acid (C16∶0) were purchased tested. The results showed that the palmitoleic acid had moderate activity against *A. tamarense*, while palmitelaidic acid and palmitic acid were almost or completely inactive (Table 3). The structure of palmitoleic acid is illustrated in Fig. 4, and the effects of different concentrations of palmitoleic acid on the growth of *A. tamarense* are illustrated in Fig. 5. The palmitoleic acid showed significant growth-inhibiting effects on *A. tamarense* at all the concentrations tested, except 10 μg/mL, and the inhibitory effects increased with the increasing concentration of the palmitoleic acid. Compared with control groups (Fig. 6a, c), all other treatment groups showed sensitivity to the palmitoleic acid, albeit to varying extents (Fig. 6d–h) after one day incubation. Algal growth was suppressed by treatment with palmitoleic acid when the concentration was higher than 20 μg/mL in the 5-day assay and almost completely inhibited at 80 μg/mL (Fig. 5). Most of algal cells aggregated and deposited at the bottom of the flasks when 40 μg/mL and 60 μg/mL of palmitoleic acid were added (Fig. 6f, g). At the concentration of 10 μg/mL, the algal cell density was reduced after two days, and although algal growth restored within 48 h, it was suppressed markedly compared with the control (Fig. 5). Fig. 6b reveals that the bacterial supernatant (cultivated for 24 h) had a significant influence on the cells of *A. tamarense*, which was similar to the palmitoleic acid treatments groups.

**Table 3:** Reported fatty acid algicide.

Algicide (fatty acid)	Target species	EC_50_ (μg/mL)	Source or reference
hexadeca-4,7,10,13-tetraenoic acid	*H. akashiwo*	1.35	[41]
octadeca-6,9,12,15-tetraenoic acid	*H.akashiwo*	0.83	
alpha-linolenic acid	*H. akashiwo*	1.13	
palmitic acid	*H. akashiwo*	79.28	[55]
	*C.marina*	29.5	
palmitelaidic acid	*H. akashiwo*	>100	
	*C.marina*	43.75	
palmitoleic acid	*H. akashiwo*	7.28	
	*C. marina*	20.31	
linoleic acid	*A. tamarense*	98.4	[51]
	*A. taylori*	72.47	
	*C. marina*	22.35	
	*H. akashiwo*	1.91	
Oleic acid	*Chlorella sp.*	12.4	[56]
	*P. simplex*	19.8	
Glycerolipids (tetraenoic acid)	*A. catenella*	20*	[57]
	*K. mikimotoi*	20*	
	*H. akashiwo*	20*	
fatty acids	*Chlorella*	10000*	[44]
nonanoic acid	*M. aeruginosa*	10*	[42]
butyric acid	*M. novacekii*	8.8*	[58]
palmitoleic acid (cis-C16∶1 n-7)	*A. tamarense*	20*	In this study
palmitelaidic acid (trans- C16∶1 n-7)	*A. tamarense*	>100*	In this study
palmitic acid	*A. tamarense*	>100*	In this study

* Values are MIC, rather than EC50.

**Figure 7:**
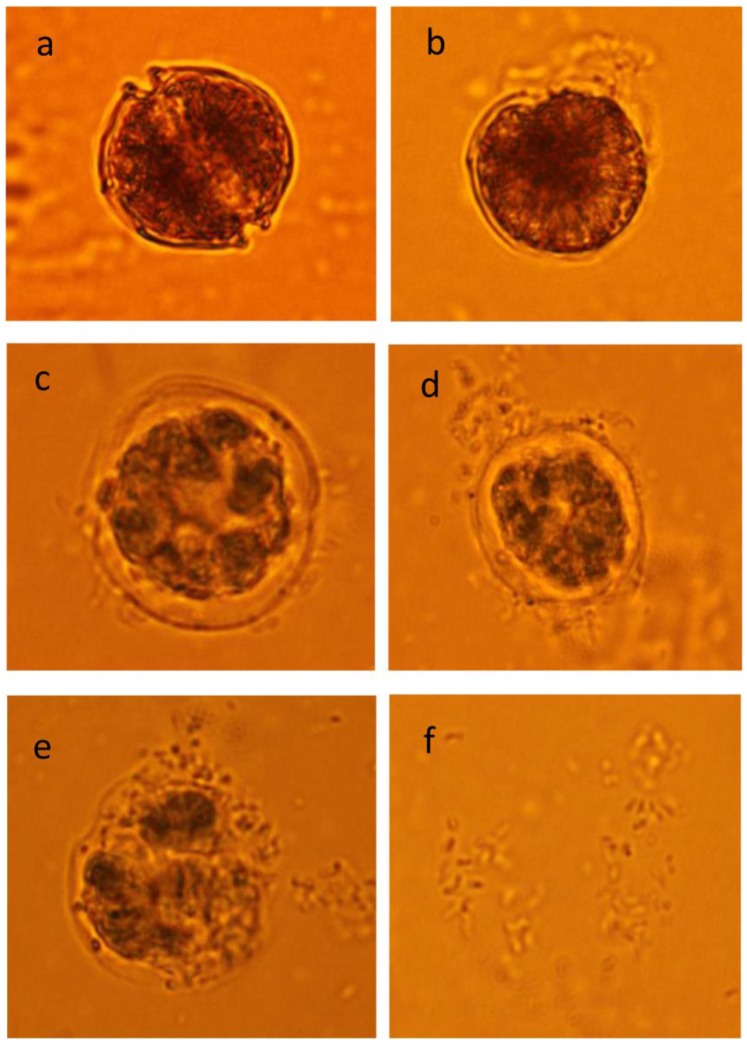
The effects of palmitoleic acid on *A. tamarense* (40 μg/mL). (a) control cells of A. *tamarense* treated with AC1 broth; (b) cells treated with palmitoleic acid on the first day; (c) cells treated with palmitoleic acid after 2 d; (d) cells treated with palmitoleic acid after 3 d; (e) cells treated with palmitoleic acid after 4 d; (f) cells treated with palmitoleic acid after 5 d; (×100).

The effects of palmitoleic acid on the lysis of *A. tamarense* cells were observed continuously for 120 h under light microscopy. Fig. 7 shows the morphological changes of the *A. tamarense* cells, which were treated with palmitoleic acid at a concentration of 40 μg/mL. Fig. 7a shows the normal cells in the control, with integrity of the cell membrane and cell wall. Compared with control (Fig. 7a) the cells initially shrank and then gradually became wrinkled with the cell wall and the cytoplasm showing marked separation after the 48 h treatment (Fig. 7c). Degradation of the organelles and disintegration of the cell wall could be observed after 72 h of treatment (Fig. 7d). With increased exposure time, the *A. tamarense* cells lysed, cellular components decomposed and were released from the cell (Fig. 7e). After 120 h, the color of the algal culture became milky white and most cells were degraded into debris (Fig. 7f).

### Effect of Palmitoleic Acid on the Growth of other Algal Species

The algicidal range of palmitoleic acid is illustrated in Table 4, where it shows strong algicidal activity against *A. tamarense* (ATGD98-006) and *A. tamarense* DH01(AT); some algicidal activity against *Alexandrium minutum* (AMTW), *Dunallella salina* (DS); while *Cyanobacteria* (BA), *Chlorella autotrophica* (CA), *Heterosigma akashiwo* (HA), *Chattonella marina* (CM), *Phaeodactylum tricornutum* (PT), *Asterionella japonica* (AJ), *Nannochloropsis* (NC) and *Phaeocystis globosa* (PG) showed no response to palmitoleic acid.

**Table 4:** Effects of palmitoleic acid on other microalgae.

		Algicidal activity of	
	Target species	palmitoleic acid	Control
Dinoflagellate	*Alexandrium tamarense* DH01(AT)	++	−
	*Alexandrium minutum* (AMTW)	+	−
	*Alexandrium tamarense* (ATGD98-006)	++	−
Chlorophyta	*Chlorella autotrophica* (CA)	−	−
	*Dunallella salina* (DS)	−	−
Bacillariophyta	*Asterionella japonica* (AJ)	+	−
	*Phaeodactylum tricornutum* (PT)	−	−
Cyanobacteria	*Cyanobacteria* (BA)	−	−
Chrysophyta	*Nannochloropsis* (NC)	−	−
	*Phaeocystis globosa* (PG)	−	−
Xanthophyta	*Heterosigma akashiwo* (HA)	+	−
	*Chattonella marina* (CM)	−	−

“++” indicates more than 70% cell mortality; “+” indicates moderate mortality, i.e. between 30 and 69%; “−” indicates less than 30% cell mortality. Control: algal cultures with the same volume of the dimethyl sulfoxide added.

### Ultrastructural Changes of *A. tamarense* Observed using Transmission Electron Microscopy (TEM)

**Figure 8:**
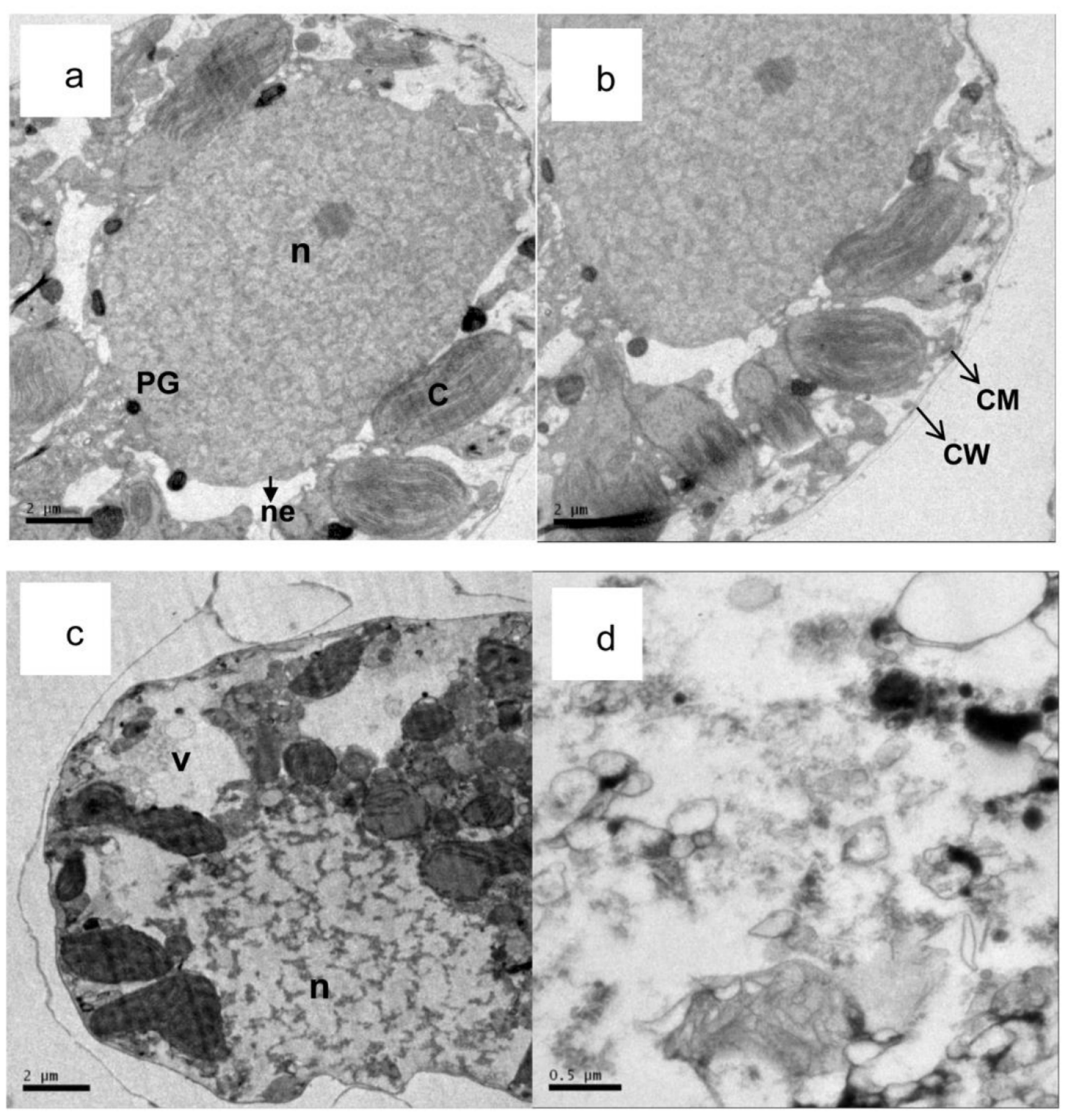
Transmission electron micrographs of the lysing process in *A. tamarense* treated with palmitoleic acid (40 μg/mL). (a,b) control cells of A. *tamarense* (×1000); (c) a damaged A. *tamarense* cell after 2 d, (×1000); (d) a damaged A. *tamarense* cell after 5 d, (×1000). (CM: cell membrane; CW: cell wall; V: vacuole; n: nucleus; C: chloroplast; ne: nuclear envelope and PG: plastoglobule.).

**Figure 9:**
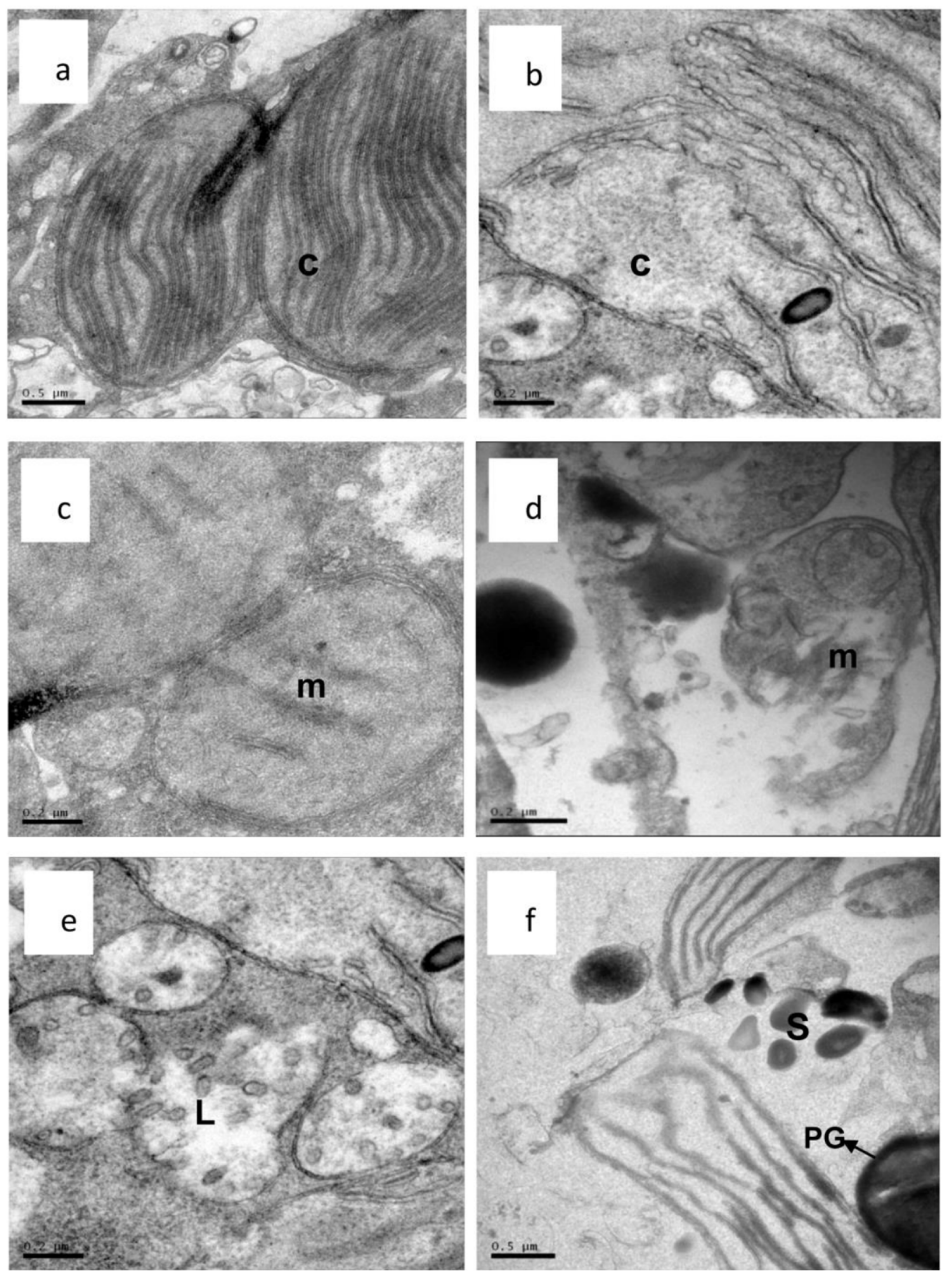
Ultrastructure of *A. tamarense* cells stressed by palmitoleic acid (40 μg/mL). (a,b) an intact chloroplast and a damaged chloroplast after 3 d (×50,00); (c,d) an intact mitochondrium and a damaged mitochondrium after 3 d (×15,000); (e,f) a damaged lysosome after 3 d and randomly distribution of the starch grains and plastoglobule (×15,000). (C: chloroplast; m: mitochondria; L: lysosomes; PG” plastoglobule and S: starch grains.).

The complex cytoplasmic organization of the microalga is often investigated using TEM. As shown in Fig. 8a and b, the intact cell wall and plasma membrane of *A.tamarense* were distinct. The plasma membrane enveloped the cytoplasm, which contained ribosomes, pyrenoglobuli and other cell organelles, such as mitochondria, chloroplasts, a nucleus and lysosomes. The shape of the mitochondria in the algal cells was predominantly oval, and they had distinct tubular cristae (Fig. 9c). Both the chloroplasts and mitochondria had a double membrane (Fig. 9a and 9c). The algal cell damage caused by exposure to palmitoleic acid is illustrated in Fig. 8c, 8d, 9b, 9d, 9e and 9f. When compared with the control (Fig. 8b), distinct plasmolysis and vacuolization occurred in algal cells treated with palmitoleic acid after 2d, and the cell wall and cell membrane were partly dissolved at the surface (Fig. 8c). The lack of an intact membrane facilitated the entry of palmitoleic acid into the cells, where it damaged other organelles, and chloroplasts, while mitochondria and nucleus were deformed. The damage was more severe when the exposure time was prolonged to 3d. Striking modifications, in comparison with the controls (Fig. 9a and 9c), appeared in the chloroplasts and mitochondria. The structure of the thylakoids was completely destroyed (Fig. 9b), and the membranes of the mitochondria were partly disintegrated, and the cell became vacuolar in the center. In addition, the cristae were somewhat distorted (Fig. 9d). Also the number of lysosomes and starch grains increased significantly (Fig. 9e and 9f) and the pyrenoglobuli randomly distributed in the cells (Fig. 9f), both remarkable changes. Finally, many cell organelles were totally disintegrated (Fig. 8d).

## Discussion

The algicidal substances reported in the literature are an array of secondary metabolites produced by algicidal bacteria, which mainly include biosurfactant [35], proteins [24], [36], amino acid [37], antibiotic [38], [39] and pigments [40]. Previous studies indicate that a newly identified member of the γ-proteobacteria, *Hahella chejuensis* KCTC 2396 releases an antibiotic prodigiosin that exhibits inhibition effects on *Cochlodinium polykrikoides* [40]. A glycolipid-type compound isolated from *Bacillus subtilis* C1 shows algicidal activity against *Microcystis aeruginosa*, and the anti-algal allelochemicals isolated from *Ulva fasciata* are mainly polyunsaturated fatty acids and derivatives [41]. The bacterium *Vibrio* sp. BS02 had a strong algicidal effect on the toxic dinoflagellate *A. tamarense.* Our previous studies showed that the BS02 led to rapid lysis of algal cells by secreting a stable secondary metabolite. The metabolite could easily be extracted using EA, indicating that the algicidal substance was hydrophobic with moderate polarity, as well as non proteinaceous. After a series of treatments involving column chromatography and TLC we obtained an oily white component, which we identified as a fatty acids (palmitoleic acid).

Fatty acids are often found to be anti-algal allelochemicals for microalgae. For instance, four fatty acids and polyphenols in *Myriophyllum spicatum*, are found to exhibit inhibitory effects on algae [42]. The polyunsaturated fatty acid EPA and the anthropogenic DnOP, which have algicidal activity against the toxic dinoflagellate *Cochlodinium polykrikoides*, were isolated from the red alga *Corallina pilulifera* [43]. Our study is the first to report palmitoleic acid as an anti-algal compound isolated from *Vibrio* sp. BS02. Algicidal activity showed that palmitoleic acid significantly inhibited the growth of *A. tamarense* with an EC_50_ of 40 μg/mL. So far many fatty acids have been reported to have the ability to inhibited alga growth (shown in Table 3). These results demonstrate that unsaturated fatty acids may play an important role in limiting the growth of alga [44].

Laboratory studies indicate that bacteria and their algicidal compounds may exhibit a broad range of specificity [40], [45], [46], while, some algicidal bacterial compounds are only effective on a specific genus or species of alga. For example, host range assays of the algicidal bacterium HYK0203-SK02 reveal that it strongly inhibits the growth of *Microcystis aeruginosa*, but stimulates the growth of the diatom *Cyclotella* sp. [47]. Additionally, 5,8,11,14,17-eicosapentaenoic acid an algicidal compound isolated from a red alga shows algicidal activity against *Skeletonema costatum*, *Chaetoceros curvisetus* and *C.polykrikoides*, but not against *Prorocentrum minimum* and *Scrippsiella trochoidea* [43]. In our study we found that the palmitoleic acid has limited algicidal effects on target algal species, such as *A. tamarense* DH01(AT), *A. minutum* (AMTW), *Alexandrium tamarense* (ATGD98-006), *Asterionella japonica* (AJ) and *Heterosigma akashiwo* (HA). Such results indicated that palmitoleic acid might be a security bio-agent for future use in controlling specific target algal blooms.

There are several types of morphological changes that take place in algal cells treated with different kinds of the algicidal compounds [48], [49]. The volatile compounds from cyanobacteria can cause shrinking and then wrinkling, and the terpenoids contacted directly cause stripping, while the basic amino-acids cause swelling and then collapse [50]. Our preliminary study using light microscopy revealed that the *A. tamarense* cells treated with palmitoleic acid initially shrank and then gradually wrinkled with the cell walls and cytoplasm showing marked separation. Finally, all the organelles and cell walls were disintegrated. These morphological changes were similar to those caused algal cells treated with volatile compounds.

Some free fatty acids are reported to be toxic to phytoplankton, and the toxic effects are multiple [51], [52]. In order to further elucidate the lytic mechanism of palmitoleic acid, the ultrastructural changes of the algal cells were observed. From the results we found that following palmitoleic acid treatment the first obvious changes in the algal cells occurred in the cell wall and cell membrane. Once the integrity of the cell wall and the plasma membrane are damaged, palmitoleic acid and other substances get inside the algal cells, and the ultrastructure of cell organelles such as mitochondria, chloroplasts and nuclei would consequently be damaged. The chloroplasts are the site of photosynthesis, and so ultrastructural changes in the chloroplasts can significantly inhibit both the growth and photosynthetic activity by decreasing photon absorption, electron transport and the reaction center of PSII in the alga [53]. As the exposure time was prolonged, the number of lysosomes increased, the function of the organelles became completely damaged, and the cells totally lysed.

The activity of a number of fatty acids in inhibiting the growth of algae are reported (Table 3). Kamaya *et al*. (2003) investigated the activity of the fatty acids C14–C18 using the growth inhibition test on the green alga *S. capricornutum* [54]; Alamsjah *et al*. (2009) discuss the algicidal activity of the polyunsaturated fatty acids, such as hexadeca-4,7,10,13-tetraenoic acid C16∶4 n-3, octadeca- 6,9,12,15 -tetraenoic acid C18∶4 n-3, α-linolenic acid C18: 3 n-3 and linoleic acid C18∶2 n-6 [55]. The results of these studies demonstrate not only that fatty acid toxicity seems to be affected by the total number of carbons and double bonds but also by the position and configuration of the double bonds in the molecule. In our study we speculated that the most probable lysis mechanism is that the fatty acid could change the osmotic pressure of the algal culture, which may lead to the creation of algal cell plasmolysis, and might even destroy the cell wall and cell membrane. Once the integrity of the cell wall and cell membrane was damaged, internal organelles such as mitochondria, chloroplasts and the nucleus are destroyed, eventually leading to total algal cells lysis.

## Conclusions

Palmitoleic acid an algicide isolated from the algicidal bacterium *Vibrio*sp. BS02 was first reported. The algicidal activity test shows that palmitoleic acid has an EC_50_ value of 40 μg/mL against *A. tamarense*. Host range analysis revealed that palmitoleic acid appeared to be somewhat species-specific. Evidence of moderate algicidal effects of palmitoleic acid on harmful algae and the lysis mechanism involved was also provided. These results might provide a new pathway for controlling HABs using fatty acids.
